# Reducing negative affect and increasing rapport improve interracial mentorship outcomes

**DOI:** 10.1371/journal.pone.0194123

**Published:** 2018-04-04

**Authors:** Jordan B. Leitner, Özlem Ayduk, C. Malik Boykin, Rodolfo Mendoza-Denton

**Affiliations:** Department of Psychology, University of California, Berkeley, Berkeley, California, United States of America; Washington State University, UNITED STATES

## Abstract

Research suggests that interracial mentoring relationships are strained by negative affect and low rapport. As such, it stands to reason that strategies that decrease negative affect and increase rapport should improve these relationships. However, previous research has not tested this possibility. In video-chats (Studies 1 and 2) and face-to-face meetings (Study 3), we manipulated the degree of mutual self-disclosure between mentees and mentors, a strategy that has been shown to reduce negative affect and increase rapport. We then measured negative affect and rapport as mediators, and mentee performance (quality of speech delivered; Studies 1 and 3) and mentor performance (warmth and helpfulness; Studies 2 and 3) as key outcomes. Results revealed that increased self-disclosure decreased negative affect and increased rapport for both mentees and mentors. Among mentees, decreased negative affect predicted better performance (Studies 1 and 3). Among mentors, increased rapport predicted warmer feedback (Studies 2 and 3). These effects remained significant when we meta-analyzed data across studies (Study 4), and also revealed the relationship of rapport to more helpful feedback. Findings suggest that affect and rapport are key features in facilitating positive outcomes in interracial mentoring relationships.

## Introduction

Research suggests that mentorship plays a critical role in personal and professional development. For instance, individuals who receive positive mentorship show more positive attitudes towards their career and earn higher salaries [1–6]. The benefits of mentorship also extend to the mentors themselves, as evidenced by research showing that people who provide mentorship exhibit better performance [3]. Notably, mentorship has been linked to positive outcomes across an array of contexts, including informal peer-to-peer relationships [7].

As such, it is important to understand how to maximize the potential benefits of mentoring relationships. This is particularly so within interracial mentoring contexts, given research showing that mentorship quality is poorer in interracial vs. same-race mentoring relationships [8, 9]. Furthermore, as a function of their under-representation in the professoriate as well as management [10,11], racial minorities (heretofore “minorities”) may frequently receive mentorship from Whites. Though previous work has identified negative affect and low rapport as two characteristics of ineffective interracial mentoring relationships, little is known about whether these relationships could be improved by implementing strategies that decrease negative affect and increase rapport. Across 3 studies, we tested whether mentee performance and mentor feedback in interracial mentoring dyads could be improved by a self-disclosure procedure that has been shown to decrease negative affect and increase rapport.

### Negative affect

Research suggests that one factor that disrupts interracial mentoring relationships is that minority mentees experience negative affect (i.e., unpleasant subjective feelings or mood) when they receive feedback from White mentors. For instance, minorities often worry that they are the target of negative racial stereotypes [12, 13] and interpret Whites’ critical feedback as racially motivated [14]. Furthermore, the anticipation of being targeted by negative stereotypes has been linked to negative arousal at both psychological and physiological levels [15–18].

Evidence suggests that minority mentees’ negative affect, in turn, diminishes their performance. For instance, research on stereotype threat suggests that negative arousal plays a role in the link between stereotype threatening contexts and underperformance [19, 20]. Additionally, theoretical frameworks posit that experiencing negative arousal increases efforts to suppress such arousal, and these suppression processes disrupt performance [15]. Consistent with this view, suppression of stereotype-related thoughts [21] has been linked to underperformance in stereotype threatening contexts.

Interracial mentoring quality may also be diminished when White mentors experience negative affect as they provide feedback to minority mentees. Supporting this possibility, research indicates that majority-group individuals in intergroup interactions often experience distress [22], and feel anxious that they will be perceived as prejudiced [23]. As feelings of distress can, in turn, promote excessive self-focus that disrupts intergroup interactions [24–26], mentors who experience more negative affect during mentoring interactions may provide poorer mentorship (e.g., provide feedback that is less warm and helpful). Thus, it would be important to understand whether strategies that reduce negative affect would improve outcomes for mentees and mentors in interracial relationships.

### Rapport

In addition to experiencing negative affect, mentees and mentors might often feel a low degree of rapport with one another. Here, rapport refers to the degree to which an individual feels a sense of interconnectedness and closeness with another person. Suggesting that rapport might be lower in interracial (vs. same-race) mentoring relationships, people underestimate the degree to which other-race individuals are interested in developing intergroup relationships [27, 28]. Moreover, members of different racial groups often have different life experiences [29], and thus might believe that they have little in common.

Low rapport might influence the behavior of both mentees and mentors in ways that limit the productivity of the relationship. For instance, mentees who feel low rapport with their mentors may avoid seeking constructive feedback from their mentor, and in turn, show lower performance. Mentors who feel low rapport with their mentees may likewise feel less committed to providing constructive, helpful feedback to their mentee. Consistent with these possibilities, previous theoretical frameworks posit that mentoring relationships suffer when mentors and mentees do not identify with one another [30]. Thus, it would be important to understand whether strategies that increase rapport would improve outcomes for mentees and mentors in interracial relationships.

### Decreasing negative affect and increasing rapport

Given evidence that negative affect and low rapport negatively affect mentoring relationships, and in particular interracial mentoring relationships, an open question is whether decreasing negative affect and increasing rapport among mentor-mentee dyads would improve performance outcomes for both members of the dyad. One potential strategy for decreasing negative affect and increasing rapport in mentoring relationships is to have mentor-mentee dyads mutually self-disclose. Self-disclosure refers to the sharing of personal information (e.g., experiences, fears, goals) with another individual [31]. Supporting the link between self-disclosure and decreased negative affect, research has demonstrated that self-disclosure gives people an opportunity to self-affirm personal values and skills, which in turn can mitigate negative affect [32, 33], including in interracial contexts [34–37]. Similarly, for rapport, individuals who self-disclose with a relationship partner consistently experience more positive attitudes towards, and feel a sense of inter-connectedness with, their partner [38–40]. Furthermore, mentees who self-disclose with their mentors show greater relationship satisfaction [41]. As discussed above, such increased rapport might contribute to both better performance among mentees and more effective mentorship among mentors.

### Current research

Based on the research reviewed above, we hypothesized that, in interracial mentoring relationships, decreasing negative affect and increasing rapport (through mutual self-disclosure) would improve mentee performance and mentor feedback. Studies 1 and 2 were conducted via video chat and Study 3 used face-to-face dyads.

Even though all participants in Studies 1 and 2 thought they were interacting with a real-life partner, the video chat interface allowed us to use pre-recorded stimuli instead of actual partners, giving us experimental control over the stimuli that participants saw. Study 1 focused on mentees’ performance; as such, participants interacted with pre-recorded “mentors.” Study 2 focused on mentors’ performance; as such, participants interacted with pre-recorded “mentees.” Mentees’ performance (Study 1) and mentors’ feedback (Study 2) were later rated by independent judges. In Study 3, we replicated and extended the findings of Studies 1 and 2 in a face-to-face dyadic mentoring context. Finally, in Study 4, we meta-analyzed data across these experiments. All studies were approved by the University of California, Berkeley Institutional Review Board (protocol # 2013-06-5421).

## Study 1

In Study 1, minority participants from online participant sites adopted the role of a mentee, and were instructed to prepare and give a speech. We used an established self-disclosure manipulation to reduce negative affect and increase rapport between mentees and mentors. To isolate mentee-level processes, independent from any mentor effects, we used pre-recorded videos of mentor actors, who were either Black or White. By using the same pre-recorded videos of mentors across the experimental conditions, we ensured that any effects across conditions were due to the content of the manipulation, as opposed to different mentors appearing in the different self-disclosure conditions. Independent judges rated the quality of participants’ speeches so that we could determine whether decreased negative affect or increased rapport (that stemmed from self-disclosure) predicted better speech performance.

### Methods

#### Participants

We recruited individuals from Mechanical Turk and Qualtrics’ Participant Panel to participate in a 2(self-disclosure: low vs. high) x 2(mentor race: White vs. minority) between-subjects design. We targeted participants who self-identified as Black or Latino/a. The sample included 155 participants (67 male; *M*_age_ = 28.06, *SD*_age_ = 8.30; 82 Black, 70 Latino/a, 3 missing race data). We analyzed Black and Latino/a participants together, given that both groups have been targeted by negative stereotypes, and have been considered minorities in many academic and professional domains [34, 35]. Additionally, effects were not moderated by whether participants were Black or Latino/a. Twenty-two additional participants began the study, but their video chats disconnected before completing all measures, and were thus omitted from analyses. All research was approved by the University of California, Berkeley Institutional Review Board (protocol # 2013-06-5421). All participants in all studies gave written informed consent and were at least 18 years of age.

#### Procedure

After completing a series of baseline measures (see below), participants were directed to a video chat room (gruveo.com) where they were connected with a live experimenter. Here, the experimenter explained that the purpose of the study was to examine how people from different racial groups communicate over the Internet and evaluate one another.

Participants were instructed to adopt the role of a “student,” and were told that later in the study, they would perform a speech that would be evaluated by a live mentor (who, in reality, was a video feed of the same-sex, White or Black pre-recorded actor; see below). Participants were informed that their mentor was a student at the University of California, Berkeley. In order to emulate the experience of a novel mentoring dyad, no other background information was given about the mentor.

**Self-disclosure manipulation.** Participants were then assigned to the high or low self-disclosure condition. In the high self-disclosure condition, participants and their mentor took turns responding to a series of prompts that encouraged escalating levels of self-disclosure. For instance, an early prompt was “If you could invite anyone over for dinner, who would it be?” while a later prompt was “What has been your biggest disappointment in life?” Participants and their mentor answered these prompts for approximately 30 minutes. Self-disclosure prompts were adopted from previous research [34,39] though we omitted prompts that might be considered inappropriate in mentoring conversations (e.g., prompts that were explicitly about romantic relationships).

As a control manipulation, the low self-disclosure condition required that participants take turns reading passages from novels to each other for approximately 30 minutes. This task served as an appropriate low self-disclosure condition since it required that participants and their “mentors” communicate in a structured environment and take turns speaking, but without disclosing any personal information.

In both the high and low self-disclosure tasks, participants viewed their mentor’s “webcam video” (the pre-recorded video) when the mentor was speaking. However, when participants were speaking, they did not see a video of their mentor. Instead, participants saw a black screen that indicated, “Your webcam is now being broadcast to your mentor.” We implemented this design feature since the mentors were prerecorded, and thus would not have shown natural non-verbal responses (e.g., head nods, smiles) when participants were speaking.

**Speech.** Participants were instructed to prepare a speech on the topic of “Why I am qualified for my dream job.” Participants were given 3 minutes to prepare, and were informed that following the speech, their mentor would evaluate them over video chat. After the preparation period, participants delivered their speech while viewing the screen indicating that their webcam was being broadcast to their mentor. Additionally, participants were presented with a clock that counted down from 5:00 to indicate how much time had elapsed in the speech. Participants were instructed to try to speak for 5 minutes. Participants did not receive an evaluation after their speech. Speeches were recorded with screen-capture software. No participants expressed suspicion to the experimenter regarding whether they were truly connected to their mentor. Following the study, participants were fully debriefed.

#### Pre-recorded mentor stimuli

Prior to the study, we generated and pre-tested stimuli of mentor videos that varied in race (White vs. minority), but were equivalent on other dimensions important to the interaction. To that end, we videotaped actors performing scripted answers to prompts from the high and low self-disclosure tasks (see below). Out of this pool, we selected four actors (one Black male, one White male, one Black female, and one White female). We tested whether these actors would receive equivalent ratings from naïve perceivers. Specifically, 148 participants (91 male, 57 female; *M*_age_ = 32.56, *SD*_age_ = 10.52; 83 White, 65 non-White) from Mechanical Turk who were not in the main study viewed videos of one of the confederates answering a subset of the high self-disclosure prompts. Participants then rated the degree to which the actor was warm and friendly on separate 7-point scales, *α* = .82. We averaged responses to these two items. A one-way ANOVA indicated that ratings of warmth/friendliness were equivalent for the Black (*M* = 5.86, *SD* = 1.00) and White (*M* = 6.08, *SD* = .61) actors, *F*(1, 146) = 2.32, *p* = .130. Actor race did not significantly interact with actor gender, rater gender, or rater race, *p*s > .096. Thus, Black and White actors were perceived to be equivalent on warmth/friendliness.

Additionally, participants viewed videos of one of the actors reading a passage from the low self-disclosure task (see below), and rated the degree to which the actor read clearly and read well on separate 7-point scales, *α* = .89. We averaged responses to these items to index perceived reading ability. Perceived reading ability ratings were equivalent for the Black (*M* = 5.48, *SD* = 1.07) and White (*M* = 5.44, *SD* = 1.07) actors, *F(*1, 146) = .06, *p* = .801. Actor race did not interact with actor gender, rater gender, or rater race, *p*s > .059. Thus, Black and White actors were perceived to be equivalent on reading ability.

Furthermore, we examined whether perceptions of overall positivity were equivalent for Black and White actors. Specifically, 143 separate participants from Mechanical Turk (86 males; *M*_age_ = 34.21, *SD*_age_ = 10.67; 91 White, 52 non-White, 22 missing race data) viewed videos of each actor performing a scripted description of their professional qualifications. Participants then rated the degree to which the actor was intelligent, persuasive, confident, sincere, organized, attractive, likeable, articulate, comfortable, calm, and clear, which were averaged to yield a single measure of positivity *α* = .90. Positivity ratings were equivalent for Black (*M* = 4.60, *SD* = 1.00) and White (*M* = 4.33, *SD* = 1.17) actors, *F*(1, 141) = 2.25, *p* = .136. Actor race did not significantly interact with actor gender, rater gender, or rater race, *p*s > .071. Thus, Black and White actors were perceived to be equivalent on ratings of overall positivity.

#### Manipulation check: Degree of disclosure

To determine the effectiveness of the manipulation, participants used a 1(strongly disagree) to 7(strongly agree) scale to respond to the items: “I shared a lot of personal information with my mentor” and “my mentor shared a lot of personal information with me.” Responses were positively related, *r* = .65, *p <* .001. We averaged responses across these items as our manipulation check.

#### Mediators: Affect and rapport

**Negative affect**. Before being directed to the video chat room, participants were directed to an online survey, where they indicated the degree to which they felt sad, hopeless, discouraged, angry, resentful, annoyed, fatigued, worn out, exhausted, vigorous, lively, and cheerful on a 1(not at all) to 7(extremely) response scale. We administered these items since they have been shown to capture state changes in emotions across situations [42]. We coded responses so that higher values indicated more negative affect (i.e., vigorous, lively, and cheerful were reverse-coded), and averaged responses, *α* = .89.

Immediately following the speech, participants again completed a measure of negative affect (described above), *α* = .88. While this measure was administered following the speech, we conjectured that that it might provide insight into participants’ negative affect that emerged in anticipation of, and during, the speech.

**Rapport.** To capture the degree to which participants felt rapport with their mentor, participants completed two measures that mapped our conceptualization of rapport. First, participants completed 13 items from the Toronto Empathy Questionnaire [43] using a 1(strongly disagree) to 7(strongly agree) response scale. Items were modified to be specific to the mentor in the study (e.g., “I find that I am ‘in tune’ with my mentor’s moods”), *α* = .84. Responses were averaged, such that greater values indicated greater empathy.

Second, participants completed an 8-item scale that assessed anticipated mutuality with the mentor (e.g., “If I were to meet my mentor, I think we would have a shared flow of thoughts and feelings”). This scale included 6 items that were modified from a high-quality connections measure, employed in previous work [44]. Additionally, we included the items: “I would be interested in meeting my mentor in a face-to-face interaction,” and “I think my mentor would want to meet me in a face-to-face interaction.” Participants’ responded on a 1(strongly disagree) to 7(*strongly agree*) scale. We averaged responses across these 8-items, where greater values indicated that participants anticipated that they would feel a greater connection with their mentor in a face-to-face meeting, α = .85.

The empathy and anticipated mutuality scale were intercorrelated, *r* = .43, *p* < .001. As such, for parsimony, we averaged participants’ scores across these measures to yield a single score of participants’ perceived rapport. See S1 File for analyses that model each rapport sub-scale independently.

#### Outcome: Speech performance

Three coders blind to condition rated videos of participants’ speeches on 3 separate dimensions: overall quality, clarity, and the likelihood that the participant would be hired for their dream job. Coders used 7-point scales, where higher values indicated better performance. Coders showed agreement on ratings of overall quality, *ICC* = .81, clarity, *ICC* = .66, and likelihood of getting hired, *ICC* = .76. We averaged coders’ responses on each dimension to create a composite for that dimension. Composites for quality, clarity, and likelihood of getting hired were intercorrelated, *ICC* = .94. Thus, for parsimony, we averaged composite ratings across all dimensions to yield a single index of speech performance.

### Results

#### Manipulation check

We conducted a 2(self-disclosure: high vs. low) x 2(mentor race: White vs. minority) ANOVA on the degree of disclosure. A main effect emerged for self-disclosure condition, *F*(1, 151) = 291.67, *p* < .001, *η*^2^ = .66, such that participants reported greater self-disclosure in the high self-disclosure (*M* = 6.15, *SD* = 1.11) vs. low self-disclosure (*M* = 2.56, *SD* = 1.47) condition. Thus, the manipulation had the expected effect on the degree of perceived self-disclosure between participants and their mentor.

#### Mediators

**Negative affect.** We conducted a 2(self-disclosure) x 2(mentor race) ANCOVA on post-speech negative affect, controlling for baseline negative affect. Due to computer error, 9 participants had missing data for baseline negative affect. A main effect emerged for self-disclosure condition, *F*(1, 141) = 8.65, *p* = .004, *η*^2^ = .06, such that negative affect was lower in the high self-disclosure (*M* = 1.97, *SD* = .76) than in the low self-disclosure (*M* = 2.30, *SD* = 1.04) condition. When baseline negative affect is omitted from the model, the main effect of self-disclosure remained significant, *F*(1, 151) = 4.50, p = .036, *η*^2^ = .03. Neither the main effect for mentor race nor the interaction was significant, *p*s > .76. Thus, increased self-disclosure predicted decreased negative affect, regardless of whether the mentor was White or minority.

**Rapport.** To determine whether self-disclosure influenced rapport, we conducted a 2(self-disclosure) x 2(mentor race: White vs. minority) ANOVA on rapport. A main effect emerged for self-disclosure condition, *F*(1, 151) = 5.75, *p* = .018, *η*^2^ = .04, such that rapport was greater in the high self-disclosure (*M* = 5.31, *SD* = .69) vs. low self-disclosure (*M* = 5.02, *SD* = .78) condition. Neither the main effect for mentor race nor the interaction was significant, *p*s > .22. Thus, increased self-disclosure predicted increased rapport, regardless of whether the mentor was White or minority.

#### Mentee performance

**Fig 1:**
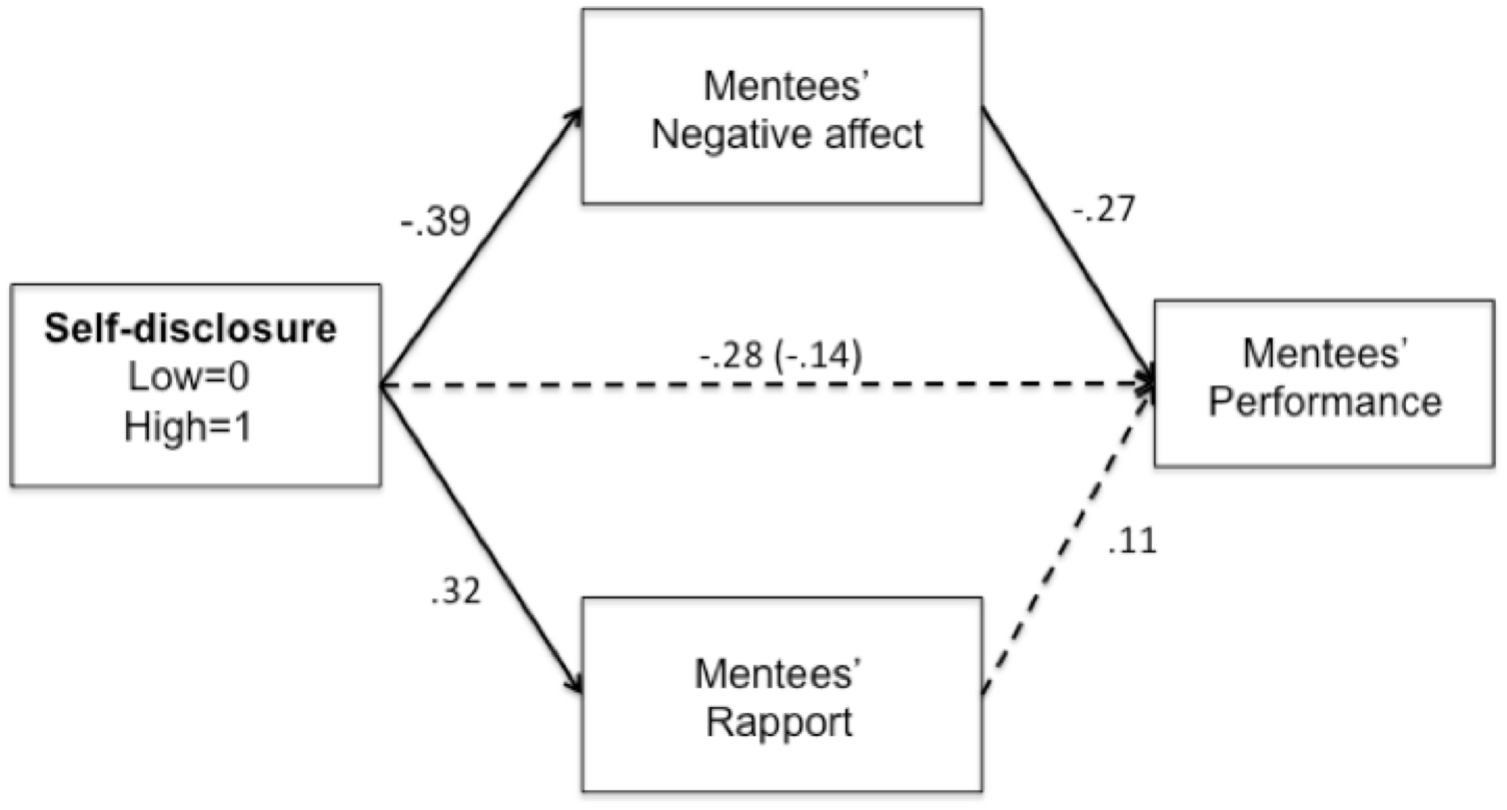
Path model predicting performance. Solid lines indicate significant paths. Dashed lines indicate non-significant paths. Coefficients are unstandardized. Parenthetical number indicates total effect coefficient.

Our principal interest was in whether the decreased negative affect and/or increased rapport (stemming from mutual self-disclosure) corresponded with improved speech performance. Accordingly, we tested for direct and indirect effects in 10,000 bootstrap resamples with PROCESS Model 4 [45]. We modeled self-disclosure condition as the predictor, speech performance as the outcome, and negative affect (controlling for baseline negative affect) and rapport as parallel mediators. To fully represent all conditions, we also entered mentor race as a covariate (it did not moderate any paths). As shown in Fig 1, increased self-disclosure predicted decreased negative affect, which in turn predicted improved performance, *ab* = .11, *SE* = .06, 95% CI [.01, .25]. Increased self-disclosure also predicted increased rapport, but the indirect path predicting performance was not significant. Thus, supporting our hypotheses, decreased negative affect was a pathway through which self-disclosure improved mentee performance. Neither the total nor direct effects were significant. Additionally, supporting our theoretical framework, the indirect effect was not significant when performance was modeled as mediator, and negative affect was modeled as an outcome.

### Discussion

To our knowledge, this is the first study to demonstrate that reducing mentees’ negative affect (via self-disclosure) improves mentee performance. We did not see independent effects of rapport on mentee performance. Although unexpected, one potential explanation is that, because mentees are the low-power individual in the dyad, their feelings of connectedness with a person giving them feedback was not as crucial for improving their performance as the tamping down of negative affect. The fact that no race effects emerged is discussed further in the General Discussion.

Although these findings demonstrated that self-disclosure in a mentoring context influenced the mentee’s experience, it remained unknown how self-disclosure might influence mentors’ behavior. Accordingly, in Study 2, we examined the link between mentors’ affect, rapport, and the quality of their feedback.

## Study 2

In Study 2, White participants from an online participant site adopted the role of a mentor and conveyed verbal feedback to their mentee. We measured mentors’ negative affect and rapport, and their feedback was independently coded for warmth and helpfulness. To isolate the effects of self-disclosure on mentors while controlling for mentee characteristics, we used the same actors as in Study 1, but presented them to participants as mentees.

### Methods

#### Participants

We recruited 144 participants (71 male; M_age_ = 29.44, SD_age_ = 10.79) from Mechanical Turk participated in a 2(self-disclosure: low vs. high) x 2(mentee race: minority vs. White) between-subjects design in exchange for payment. All participants self-identified as White. 17 additional participants began the study, but their video chats disconnected before completing all measures, and were thus omitted from analyses.

#### Procedure

Similar to Study 1, participants were directed to a video chat room, and were greeted by a live experimenter. Participants were informed that the purpose of the study was to examine how people evaluate one another over the Internet. Participants were instructed to adopt the role of a “mentor,” and that, later in the study, they would evaluate a speech performed by a live college student. Participants were instructed to think of this student as “their mentee.” In reality, participants were paired with either a Black or White same-sex pre-recorded actor. The actors in this study were identical to those in Study 1. Participants were then assigned to the high or low self-disclosure condition, following identical procedures described in Study 1.

**Speech stimulus**. Next, participants viewed their mentee performing a speech on the topic of “Why I am qualified for my dream job.” While participants were led to believe that this speech was being performed live, speeches were actually pre-recorded, and all actors performed the same speech. The speech was scripted to be moderate in quality so that it could generate both praise and criticism. Participants were informed that, following the speech, they would prove video feedback to their mentee.

**Mentor feedback.** Finally, participants were instructed to provide 5 minutes of verbal feedback that identified strengths and weaknesses of their mentee’s speech. As in Study 1, participants did not view the actor during their feedback. Instead, they viewed a screen that indicated that their webcam was being broadcast to their mentee, and showed a clock that counted down from 5:00 to indicate the amount of time that had elapsed in their feedback. We recorded participants’ evaluations with screen-capture software. No participants indicated to the experimenter that they were not actually connected to a live mentee. Immediately following the study, participants were fully debriefed.

#### Manipulation checks: Degree of disclosure

Same as Study 1.

#### Mediators: Affect and rapport

**Negative affect.** To measure baseline negative affect, participants completed a negative affect scale (described in Study 1) at the beginning of the study, α = .84. After listening to the mentee speech, but before providing feedback, participants completed the same negative affect measure described above, α = .82.

**Rapport.** After listening to the mentee speech, but before providing feedback, participants also completed a rapport measure (described in Study 1). See S1 File for analyses that model each rapport sub-scale independently.

#### Outcome: Speech performance

Three coders rated the feedback for warmth and helpfulness 1(not at all) to 7(very much) scales. Coders showed acceptable agreement for warmth, ICC = .66, and helpfulness, ICC = .82. Accordingly, we averaged coders’ ratings on each dimension.

### Results

#### Manipulation check

To determine the effect of conditions on degree of disclosure, we conducted a 2(self-disclosure) x 2(mentee race) ANOVA predicting degree of disclosure. A main effect emerged for self-disclosure, *F*(1, 140) = 537.36, *p <* .001, *η*^2^ = .79, such that responses to the manipulation check were greater in the high self-disclosure (*M* = 6.22, *SD* = .88) vs. low self-disclosure (*M* = 1.83, *SD* = 1.33) condition. Neither the effect of mentee race nor the interaction were significant, *p*s > .286. Thus, the manipulation had an effect on the degree of self-disclosure between participants and their mentee.

#### Mediators

**Negative affect.** To determine the effects of self-disclosure and mentee race on negative affect, we conducted a 2(self-disclosure) x 2(mentee race) ANCOVA on negative affect, controlling for baseline negative affect. No effects were significant, *p*s > .13, indicating that negative affect was equivalent across self-disclosure and mentee race conditions.

**Rapport.** To determine the effects of self-disclosure and mentee race on rapport, we conducted a 2(self-disclosure) x 2(mentee race) ANOVA on rapport. A main effect emerged for self-disclosure, *F* (1, 140) = 10.00, *p* = .002, *η*^2^ = .07, such that participants reported greater rapport in the high self-disclosure (*M* = 5.33, *SD* = .76), as compared to the low self-disclosure (*M* = 4.86, *SD* = 1.05) condition. No other effects were significant, *p*s > .15, indicating that greater self-disclosure increased rapport, regardless of whether mentees were minority or White.

#### Outcome: Mentor feedback

**Fig 2:**
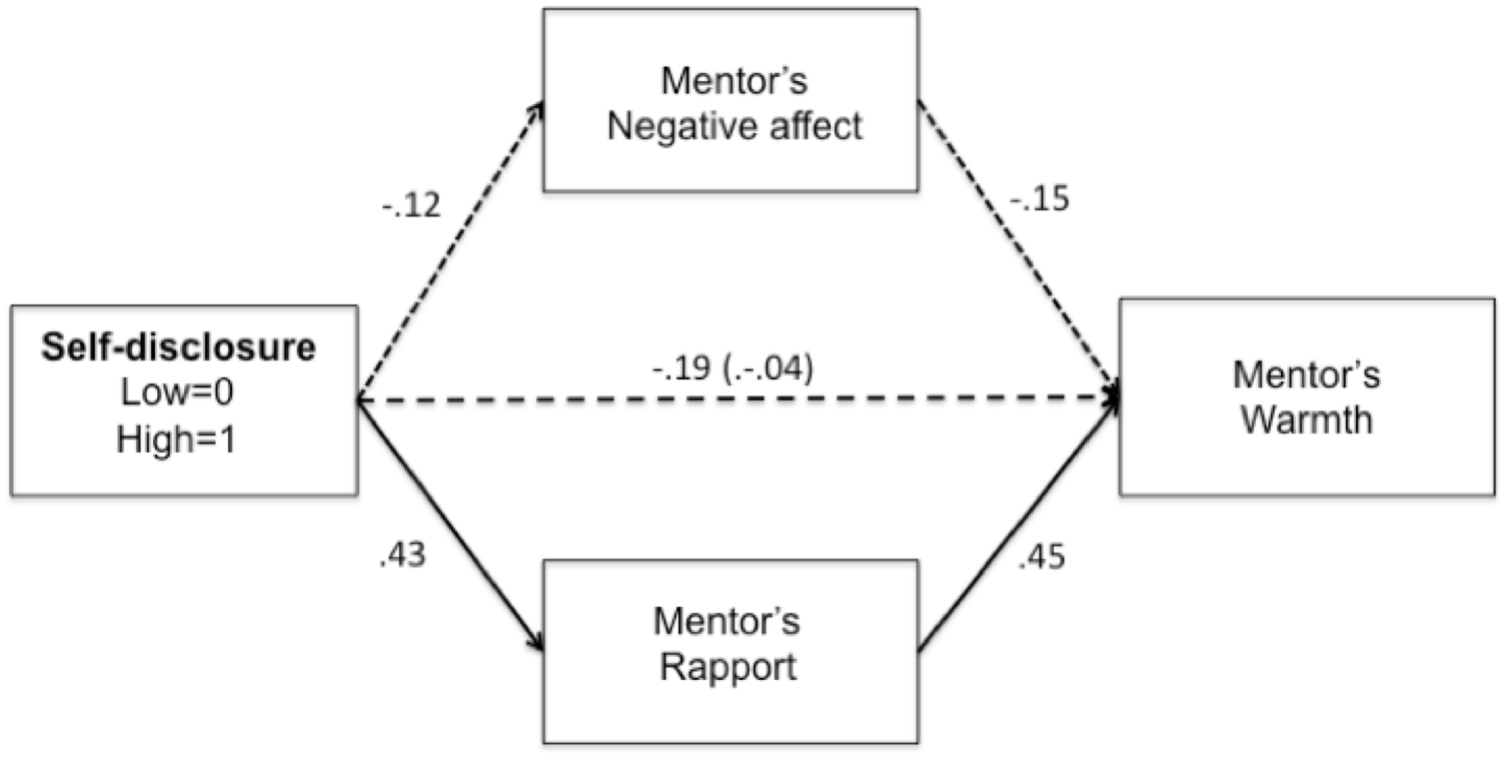
Direct and indirect effects predicting feedback warmth. Solid lines represent significant simple paths. Dotted lines represent nonsignificant simple paths. Parenthetical value indicates total effect. Coefficients are unstandardized.

To examine whether increased rapport shaped the feedback that participants conveyed to their mentee, we used PROCESS Model 4 (45) to test for direct and indirect effects in 10,000 bootstrap resamples. We modeled self-disclosure condition as the predictor, rapport as the mediator, and warmth as well as helpfulness as outcomes in separate analyses. To represent all conditions in the model, we included mentee race as a control variable (mentee race did not moderate any paths). Additionally, to represent all paths in our theoretical framework, and be consistent with analyses in Study 1, we modeled negative affect as a parallel mediator, and baseline negative affect as a control variable. Results revealed that increased self-disclosure predicted increased rapport, which in turn, predicted increased feedback warmth (Fig 2), indirect effect: *ab* = .19, *SE* = .09, 95% CI [.07, .39]. Thus, supporting our hypotheses, these findings suggest that increased rapport was a mechanism through which self-disclosure promoted warmer feedback. Contrary to our hypotheses, the indirect effect predicting helpfulness was not significant.

### Discussion

In both same-race and interracial mentoring pairs, increasing rapport (via self-disclosure) corresponded with mentors providing warmer verbal evaluations (as rated by independent coders). Taken together with Study 1, these findings suggest that mutual self-disclosure between mentees and mentors facilitates mentoring relationships by eliciting processes that promote mentee performance and increased warmth in mentor feedback.

One unexpected result was that self-disclosure was unrelated to mentors’ negative affect. A potential explanation for this null finding is that mentors did not feel apprehension about being evaluated since they were in a position of power, relative to their mentee and the interactions were online rather than face-to-face. We return to this issue in the General Discussion.

A second unexpected result was that increased rapport was unrelated to feedback helpfulness. One potential explanation for this null finding is that the online methodology reduced mentors’ motivation to provide critical and helpful feedback. To address these possibilities, and determine whether the hypothesized effects would emerge in a more ecologically valid context, we conducted Study 3.

## Study 3

Study 3 examined whether, in a face-to-face interracial mentoring context, positive affect and rapport among both mentees and their mentors affected the same outcomes we observed in Studies 1 and 2; namely, mentee performance and mentor feedback.

Study 3 was similar to Studies 1 and 2, except for the following changes: First, Study 3 was conducted in-person rather than online, and thus provided a more naturalistic setting to test the hypotheses. Second, instead of employing actors, Study 3 was a naturalistic dyad study that recruited participants to adopt the roles of mentees and mentors. An advantage of including both mentee and mentor participants is that we were able to simultaneously test whether the effects of Study 1 would replicate for mentees, and the effects of Study 2 would replicate for mentors. As a more robust test of the effects of interest, mentees gave two speeches, to allow us to determine if any effects would replicate across multiple speeches. Furthermore, to capture the negative affect that mentees experienced while they anticipated and received their mentors’ evaluations, we employed a measure that incorporated mentees’ self-reported negative affect (measured both pre-speech and post-speech) with coder-rated affect as they received their mentors’ evaluation.

### Methods

#### Participants

Participants were recruited from an introductory psychology participant pool, print advertisements, and online advertisements. We recruited 58 dyads, consisting of 116 participants (43 Latino/as, 15 Blacks, 58 Whites; *M*_age_ = 20.05, *SD*_age_ = 2.22; 70 females, 46 males). Dyads were assigned to either the high or low self-disclosure condition in exchange for payment or course credit. All participants were paired in same-sex interracial dyads consisting of one minority (Black or Latino/a) participant and one White participant. Due to equipment failure, we had missing self-report data for 6 minority participants, and missing video data for 2 dyads.

#### Procedure

**Fig 3:**
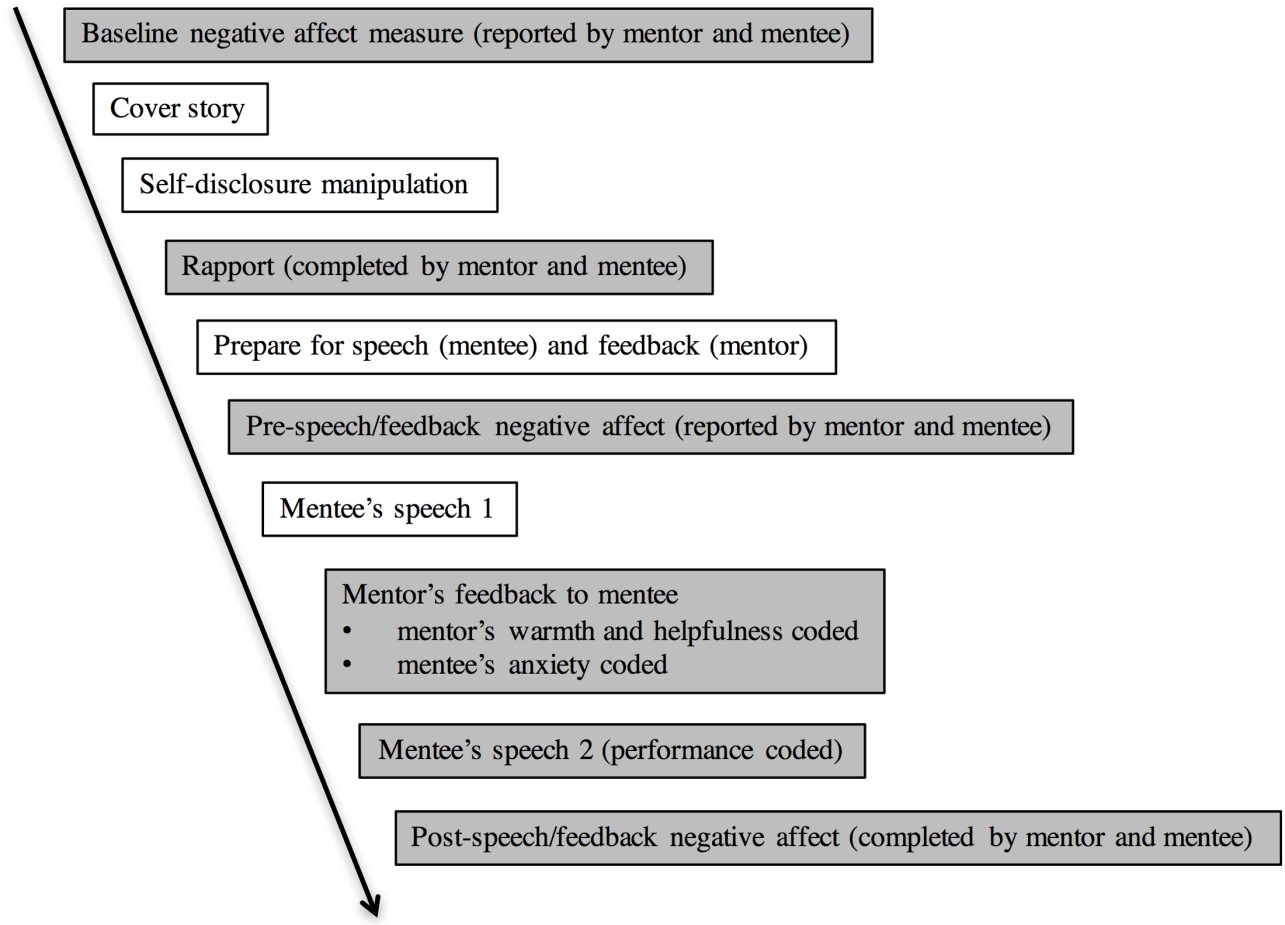
Sequence of procedures and measures in Study 3. Grey boxes indicate measures.

An experimenter guided participants through all procedures. The sequence of procedures and measures is shown in Fig 3.

**Cover story.** Participants were escorted to a common room, and seated across from one another. An experimenter explained that the purpose of the study was to examine interpersonal communication and mentorship. Although participants were not privy to role assignment, White participants were always instructed to adopt the role of the “mentor,” and minority participants were always instructed to adopt the role of the “mentee.” As part of the cover story, participants were told that this assignment process was random. Participants were then assigned to either the high or low self-disclosure condition, following procedures described in Study 1.

**Rapport.** Following the self-disclosure exercise, Participants completed the Toronto Empathy Questionnaire described in Study 1, *α* = .83. As in Study 1, items were modified to be in reference to the participant’s partner in the study (i.e., for mentees, the items referenced “your mentor”; for mentors, the items referenced “your mentee”). Second, participants completed a 6-item scale, which we generated, that more directly assessed feelings of interconnectedness with their mentor/mentee (see S1 File), *α* = .91. These two measures were positively related, mentees: *r* = .37, *p* < .001. For parsimony, we averaged scores across these two scales to yield a single score for mentee rapport, and a single score for mentor rapport. See S1 File for analyses that model each rapport sub-scale independently.

**Speech/feedback.** Mentees were then given 3 minutes to prepare a 5-minute speech on the topic of “Why I am qualified for my dream job.” As in Study 1, mentees were informed that their mentor would evaluate their speech. During this period, mentors were instructed to prepare for evaluating their mentees’ upcoming speech. Following the preparation period, mentees and mentors completed a second measure of negative affect (described in Study 1).

**Mentee speech 1.** Next, mentees were reunited with their mentors, and delivered their 5-minute speech without notes in front of their mentor. If mentees could not fill the entire 5 minutes, the experimenter provided prompts (e.g., “Identify your greatest strength and weakness”). We conceptualized the purpose of Speech 1 to be a way for the mentee to provide content that the mentor could evaluate.

**Mentor feedback to mentee.** Immediately following mentees’ speech, mentors were instructed to provide 5 minutes of verbal feedback to their mentee. Mentors were instructed to identify the strengths and weaknesses in their mentee’s speech. To assess the quality of mentors’ feedback, 3 coders rated warmth and helpfulness on 1(not at all) to 7(very much) scales. Coders showed acceptable agreement on warmth, ICC = .76, and helpfulness, ICC = .71. Accordingly, we averaged coders’ ratings to yield one measure of warmth and one measure of helpfulness.

Additionally, 3 coders used a 1(not at all anxious) to 7(extremely anxious) scale to rate mentees’ anxiety as they received this feedback. Coders showed acceptable agreement, *ICC* = .71, and we averaged coders’ ratings.

**Mentee speech *2*.** After receiving the mentor’s feedback, mentees were instructed to perform their speech a second time. To assess performance on this speech, 3 coders blind to condition made ratings on the same dimensions as in Study 1: speech quality, clarity, and likelihood of getting hired. Coders showed acceptable agreement (*ICC*_quality_ = .72, *ICC*_clarity_ = .72, *ICC*_hired_ = .68), and codes were averaged for each dimension for each speech. Additionally, the 3 dimensions were intercorrelated within participants, α = .97. As such, we averaged across the dimensions to yield a single rating for performance.

**Post-speech/feedback negative affect.** Following Speech 2, mentees and mentors again responded to the negative affect measure described in Study 1. Following this measure, participants were debriefed.

#### Analytic approach

**Mentee negative affect.** As described above, mentees reported their negative affect before Speech 1, and again after Speech 2. Additionally, coders rated mentees’ negative affect as they received their mentors’ feedback. We anticipated that these 3 measures all tapped into mentees’ apprehensiveness about being evaluated. Supporting this view, these 3 negative affect measures were positively intercorrelated, α = .68. For parsimony, we averaged participants’ scores across these 3 measures to yield a single score that reflected negative affect across the mentoring interaction. Nevertheless, findings are equivalent when we limit this negative affect composite to the 2 measures that preceded the second speech. See S1 File for analyses that model each negative affect sub-scale independently.

**Mentor negative affect.** As indicated above, mentors reported their negative affect before providing feedback and again after providing feedback. We conjectured that both these measures reflected mentors’ apprehension about providing feedback to their mentee. Supporting this view, mentors’ pre-feedback and post-feedback negative affect measures were positively related, *r* = .76, *p* < .001. For parsimony, we averaged mentors’ scores across these measures to yield a single score that reflected negative affect across the evaluation. See S1 File for analyses that model these sub-scales independently.

**Mentee performance.** As noted above, mentees performed their speech twice. We treated mentees’ performance on the second speech as the outcome measure since it occurred after the receipt of mentor feedback, and a theoretical mediator was mentees’ negative affect while receiving feedback. Nevertheless, it is possible that apprehensiveness about receiving feedback might have negatively influenced mentees’ performance on their first speech. Supporting the view, findings are equivalent if we model Speech 1 performance as the outcome, or create a composite performance measure across the two speeches. Thus, performance in Speech 2 vs. 1 as the outcome variable does not influence conclusions.

### Results

#### Mediators

**Negative affect.** Since we measured negative affect from both the mentee and mentor in each dyad, participants’ negative affect was nested within dyad. To account for this multi-level structure, we employed a mixed linear model (MLM) with restricted maximum likelihood (REML) estimation. First, we estimated the effect of self-disclosure condition (low = 0; high = 1) on negative affect, controlling for baseline negative affect. A main effect emerged, *b* = -.41, *SE* = .13, *t* = -3.09, *p <* .001, such that negative affect was lower for participants in the high self-disclosure (*M* = 2.36; *SD* = .56) vs. low self-disclosure (*M* = 2.85; *SD* = .66) condition. This effect was not moderated by whether the participant was a mentor or mentee in the dyad, *p* = .860. Thus, across mentees and mentors, higher self-disclosure corresponded with decreased negative affect.

**Rapport.** Since we measured rapport from both the mentee and mentor in each dyad, participants’ rapport was nested within dyad. Accordingly, we used MLM with REML to estimate the effect of self-disclosure condition on rapport. A main effect emerged, *b* = 1.02, *SE* = .12, *t* = 8.32, *p <* .001, such that rapport was greater in the high self-disclosure (*M* = 5.38, *SD* = .54) vs. low self-disclosure (*M* = 4.35, *SD* = .74) condition. This effect was not moderated by whether the participant was a mentee or mentor in the dyad, *p* = .87. Thus, across mentees and mentors, increased self-disclosure increased rapport.

#### Outcome measures

**Fig 4:**
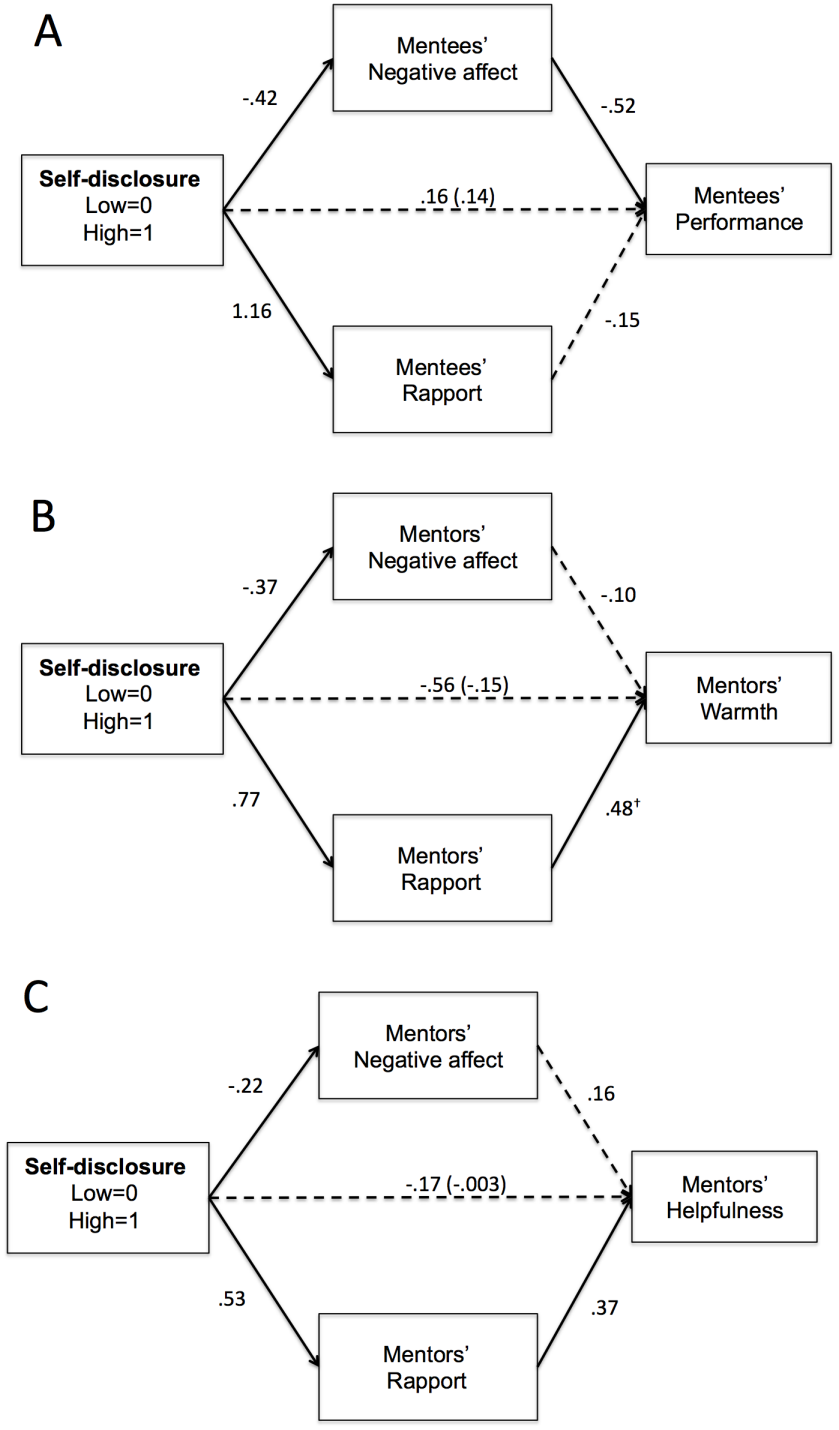
Path model predicting A: Mentees’ speech performance, B: Mentors’ feedback warmth, and C: Mentors’ feedback helpfulness. Solid lines indicate significant paths. Dashed lines indicate non-significant paths. Coefficients are unstandardized. Parenthetical number indicates total effect coefficient.

**Speech performance.** We examined whether mentees’ negative affect or rapport predicted their speech performance. Accordingly, we tested for indirect effects by employing 10,000 bootstrap estimates in PROCESS Model 4 [45]. We modeled self-disclosure condition as the predictor, mentees’ speech performance as the outcome, and the following as parallel mediators: mentees’ negative affect (controlling for baseline negative affect), and mentees’ rapport. As shown in Fig 4A, and replicating Study 1, increased self-disclosure predicted decreased negative affect, which in turn predicted increased performance, *ab* = .22, *SE* = .11, 95% CI [.05, .52]. This indirect path remained significant when we entered mentor’s negative affect and mentor’s perceived rapport as additional covariates, *ab* = .26, *SE* = .14, 95% CI [.05, .62]. Thus, supporting our hypotheses, decreased mentee negative affect was a pathway through which increased self-disclosure predicted improved speech performance. Neither the direct nor total effects were significant. Additionally, the indirect effect was non-significant when we modeled performance as the mediator, and negative affect as the outcome.

**Mentors’ feedback warmth.** Next, we examined whether mentor’s negative affect and rapport predicted the quality of feedback they provided to their mentees. We modeled self-disclosure condition as the predictor, mentors’ warmth as the outcome, and the following as parallel mediators: mentors’ negative affect (controlling for baseline negative affect) and mentors’ rapport. As shown in Fig 4B, among mentors, increased self-disclosure predicted decreased negative affect and increased rapport. Increased rapport, in turn predicted increased warmth, *ab* = .37, *SE* = .24, 95% CI [.01, .97]. This indirect effect remained significant when we modeled mentees’ negative affect and mentee’ rapport as additional covariates, *ab* = .26, *SE* = .18, 95% CI [.003, .79]. The indirect effect through negative affect was non-significant. Neither the total nor direct effects were significant.

**Mentor’s feedback helpfulness.** Finally, we estimated the same model described above, but modeled mentors’ helpfulness as the outcome variable. Contrary to our hypothesis, no direct or indirect paths were significant.

## Study 4

Studies 1 and 3 yielded a consistent picture of the effects of negative affect and rapport on mentee outcomes, and Studies 2 and 3 yielded a consistent picture of the effects of negative affect and rapport on mentor outcomes. Nevertheless, some effects varied across studies. To maximize statistical power and yield reliable estimates, we analyzed effects across these studies. Specifically, we meta-analyzed mentee effects across Studies 1 and 3, and mentor effects across Studies 2 and 3. In Studies 1 and 2, we only included data from the interracial mentee-mentor dyad conditions, as they were equivalent to the design of Study 3. Results revealed that, across Studies 1 and 3, increased self-disclosure predicted decreased negative affect, which in turn, predicted increased performance (shown in Figs 1 and 4A), *ab* = .15, *SE* = .07, 95% CI [.05, .30].

Furthermore, across Studies 2 and 3, increased self-disclosure predicted increased rapport, which in turn, predicted the provision of warmer feedback (shown in Figs 2 and 4B), *ab* = .22, *SE* = .10, 95% CI [.07, .48]. Finally, across Studies 2 and 3, increased self-disclosure predicted increased mentors’ rapport, which in turn predicted increased helpfulness, *ab* = .19, *SE* = .11, 95% CI [.03, .48] (Fig 4C). Thus, across online and face-to-face contexts, self-disclosure promoted positive interracial mentorship outcomes by decreasing negative affect among minority mentees and promoting feelings of rapport among White mentors.

### General discussion

Across three experiments, decreasing negative affect and increasing rapport (via self-disclosure) predicted better performance for mentees (Studies 1, 3, and 4) and warmer and more helpful feedback for mentors (Studies 2, 3, and 4). These findings are important, as positive mentorship is critical for personal and professional growth, and interracial mentorship is becoming increasingly common in many domains. The current results are consistent with research showing that self-disclosure promotes rapport in interpersonal [31] and intergroup [34] contexts. However, the current findings extend previous work by elucidating the specific pathways through which self-disclosure improves outcomes for mentors and mentees. Furthermore, we demonstrated this pattern of effects across both online and face-to-face contexts.

One strength of the current research is that we demonstrated the benefits of reducing negative affect for mentees and increasing rapport among mentors after only a brief meeting in novel mentoring interactions. As such, this research can inform practical interventions that are implemented in the initial stages of mentoring relationships. Nonetheless, since real life mentor-mentee relationships may provide repeated opportunities for interaction over an extended period of time, the effects of rapport and affect might generate even larger benefits than those documented in the current work. It will be important for future research to examine how the processes examined in the current work unfold over time.

One unexpected finding was that we did not observe benefits of reducing negative affect for mentors’ feedback quality, or benefits of rapport for mentees’ performance. This may have to do with the brevity of the manipulation; however, research does suggest that in status relationships, people in low-power positions may be more likely to adopt a focus on avoiding negative outcomes, while those in high power positions may be more focused on approach-related behaviors [46]. As such, it stands to reason that among mentees, reducing the negative affect that comes with evaluation may be the primary focus, whereas mentors focus more on the relational aspects of the interaction.

While self-disclosure indirectly predicted mentee performance (Studies 1 and 3), the total and direct effects of self-disclosure on performance were non-significant. As such, one question raised by the current findings is why the self-disclosure manipulation did not directly influence mentee performance. One possibility is that self-disclosure has a negative effect on performance by establishing a rapport that disrupts the ability to give and receive critical feedback. However, the fact that we do find a link between mentors’ rapport and helpfulness of feedback argues against this possibility, as does research showing that White mentors provide uncritical feedback to minority proteges in the presumed absence of rapport [47]. Another potential explanation is that high vs. low self-disclosure task was more cognitively demanding, disrupting subsequent speech performance. This explanation could also account for the non-significant direct relationship between self-disclosure and mentor helpfulness. Specifically, to the extent that higher self-disclosure was more cognitively demanding, mentors in the high self-disclosure condition may have had fewer resources to provide detailed feedback to their mentee. Future research might build on the current work by identifying the multiple pathways through which self-disclosure influences mentee and mentor behavior.

While the central purpose of the current research was to examine mentoring relationships that consisted of a minority mentee and White mentor, Studies 1 and 2 also included conditions where the mentee and mentor shared a common racial classification (i.e., Study 1 included minority-minority dyads, and Study 2 included White-White dyads). We had expected that the effects of self-disclosure would be greater in minority-White (vs. minority-minority or White-White) dyads. However, results showed that self-disclosure promoted positive outcomes to an equivalent degree across dyad types. One potential explanation for this finding centers on the nature of the low self-disclosure task (i.e., reading passages). Since this task was highly structured, its nature may have prevented the development of a common ingroup identity [48], leading to lower-than-expected positivity in the same-race dyads. Future research might continue to examine the contexts in which the racial composition of a dyad influences negative affect and rapport. The findings nevertheless suggest that decreasing negative affect and increasing rapport are effective in improving mentoring relationships in interracial contexts, perhaps to the same degree as in intra-racial contexts (c.f. [49]).

In sum, the current research established that affect and rapport are both critical components for mentoring relationships. These findings emerged in both online and face-to-face contexts. Importantly, these results might serve as a starting point for interventions that aim to improve the performance and retention of groups that have been historically under-represented. Such interventions might lead to a workforce that is capable of meeting the needs of an increasingly diverse society [50, 51].

## Supporting information

S1 File Supplementary materials. This file contains supplementary methods and analyses.
(DOCX)
