# Supplementary material for: Reducing negative affect and increasing rapport improve interracial mentorship outcomes
Source: PLoS One. 2018 Apr 4;13(4):e0194123. doi: 10.1371/journal.pone.0194123 (PMC5884483; doi:10.1371/journal.pone.0194123)
Supplement: S1 File — This file contains supplementary methods and analyses. (DOCX) [file pone.0194123.s001.docx]

**Supplementary Materials**

**Study 1**

***Rapport subscales.*** For parsimony, in the main text, we reported analyses where we indexed rapport by creating a composite of two subscales. However, we also examined effects on each subscale independently. The main effect of self-disclosure remained significant when predicting the felt mutuality scale, *F*(1, 151) = 6.87, *p =* .010, *η*^2^ = .04. This effect was in the expected direction, but non-significant, when predicting the empathy scale, *p =* .159. Thus, the effects of self-disclosure on rapport may have been driven by greater felt mutuality, as opposed to greater empathy.

***Anticipated mentor support.*** Study 1 also included a measure of anticipated mentor support. Specifically, participants used a 1(*not at all*) to 7(*very much*) scale to indicate the degree to which they anticipated that their mentor would rate them as intelligent, hardworking, persuasive, articulate, and responsible, *α* = .93. We averaged responses to these ratings. To determine whether negative affect or rapport predicted anticipated mentor support, we conducted the same indirect path model showing in Figure 2, but modeled anticipated mentor support as the outcome variable. Results revealed a significant indirect path, such that higher self-disclosure predicted decreased negative affect, which in turn predicted greater anticipated mentor support, *ab =* .20, *SE =* .11, 95% CI [.04, .46]. No other effects were significant.

**Study 2**

***Rapport subscales.*** As in Study 1, in Study 2, we indexed rapport by creating a composite of two subscales. The main effect of self-disclosure remained significant when predicting felt mutuality subscale, *F*(1, 40) = 9.79, *p =* .002, *η*^2^ = .07, as well as the empathy subscale, *F*(1, 40) = 6.12, p = .015, *η*^2^ = .04.

***Feedback valence.*** In addition to coding warmth and helpfulness, 3 raters also rated the valence of participants’ feedback on a 1(*very negative*) to 7(*very positive*) scale. We averaged the ratings of the three coders, *α* = .63. To determine whether negative affect or rapport influenced feedback valence, we modeled feedback valence as an outcome variable in the model shown in Figure 3. A significant indirect effect emerged, such that higher self-disclosure predicted increased rapport, which in turn predicted the provision of more positive feedback, *ab = .*23, *SE =* .10, 95% CI [.09, .47]. Thus, this finding suggests that higher self-disclosure increases rapport, which in turn corresponds with mentors providing more positive feedback to their mentee.

**Study 3**

***Rapport subscale items.***

Indicate the degree to which you agree with the following statements, as they relate to you’re your interaction partner (i.e., your “mentor” [“mentee”]) in today’s study.

1. I feel close with my interaction partner
2. I feel like I know who my interaction partner really is
3. I feel like my interaction partner knows the real me
4. I feel warm towards my interaction partner
5. I feel like I know a lot about my interaction partner
6. I like my interaction partner

***Rapport subscales results.*** As in Studies 1 and 2, we indexed rapport by creating a composite of two subscales. Among mentees, the main effect of self-disclosure remained a significant predictor of both empathy, *F*(1, 51) = 6.12, *p =* .017, and mentees’ feelings of closeness with their mentor, *F*(1, 52) = 68.13, *p* < .001, *η*^2^ = .57. Among mentor participants, self-disclosure was unrelated to empathy, p = .904, but it was related to their feelings of closeness, *F*(1, 56) = 56.36, *p* < *.*001, *η*^2^ = .50.

***Mentee negative affect.*** For parsimony, we created a composite of mentees’ negative affect across three time points: pre-speech (self-reported), during the receipt of feedback (coded), and post-feedback (self-reported). To determine whether one of these measures was the most critical mediator in the link between self-disclosure and performance, we revised the models shown in Figure 5. Specifically, we conducted independent analyses wherein we modeled each measure of negative affect as the mediator between self-disclosure and performance. None of these models yielded significant indirect pathways. Thus, the composite of the three negative affect measures, as compared to an individual measure, was a stronger mediator of the link between self-disclosure and mentee performance.
